# Preoperative deep venous thrombosis (DVT) after femoral neck fracture in the elderly, the incidence, timing, location and related risk factors

**DOI:** 10.1186/s12891-021-04145-4

**Published:** 2021-03-11

**Authors:** Shuai Niu, Juan Li, Yan Zhao, Dianzhu Ding, Guangwei Jiang, Zhaohui Song

**Affiliations:** 1 Department of Vascular Surgery, the General Hospital of Hebei Province, Shijiazhuang, 050000 Hebei People’s Republic of China; 2 Department of Oral and Maxillofacial Surgery, the General Hospital of Hebei Province, Shijiazhuang, 050000 Hebei People’s Republic of China; 3 School of Nursing, Hebei Medical University, Shijiazhuang, 050000 Hebei People’s Republic of China; 4 Department of Orthopaedic Surgery, the 3rd Hospital of Hebei Medical University, Shijiazhuang, 050051 Hebei People’s Republic of China

**Keywords:** Elderly, Femoral neck fracture, Deep vein thrombosis, Epidemiology, Risk factors

## Abstract

**Objective:**

To investigate the epidemiologic characteristics of deep venous thrombosis (DVT) in elderly patients with femoral neck fracture.

**Methods:**

Retrospective analysis was performed on elderly patients with femoral neck fractures admitted to two institutions from January 2016 to October 2019. Duplex ultrasonography (DUS) was used to detect DVT. Patients’ hospitalization medical records were retrieved to collect the data, which were related to demographics, comorbidities, injury and laboratory results on admission. Patients with preoperative DVT were defined as the case group and those without DVT as control group, and compared using the univariate analyses. Multivariate logistic regression analysis was used to identify the independent factors associated with DVT.

**Results:**

Totally, 980 patients met the predefined criteria and were included. Sixty-seven patients were diagnosed to have preoperative DVT, with incidence of 6.8% for overall, 1.7% for proximal and 5.1% for distal DVT. The mean time from injury to diagnosis of DVT was 6.0 ± 4.7 days (median, 5.0). Most (76.1%) patients with DVT had thrombi solely in the injured extremity, in contrast with 14.9% (10/67) in the uninjured and 9.0% (6/67) in both injured and uninjured extremity. Multivariate analysis showed chronic renal insufficiency (OR, 3.37; 95%CI, 1.57 to 7.28), current smoking status (OR, 2.42; 95%CI, 1.23 to 5.63), time from injury to DUS (OR, 1.26; 95%CI, 1.07 to 1.61) and PLT > 220*10^9^/L (OR, 1.94; 95%CI, 1.31 to 3.77) were independent factors for DVT.

**Conclusion:**

Preoperative DVT is not very prevalent following elderly femoral neck fractures, but with a certain proportion in the uninjured extremity, necessitating the more attention. These identified risk factors aid in patient counseling, individualized risk assessment and risk stratification, and should be kept in mind.

## Introduction

Femoral neck fracture is a typical osteoporotic fracture, accounting for 9–15% of overall fractures and 40–50% of the hip fractures in the elderly population [1, 2], with serious consequences that one-year mortality rate is 15.6–25.4% and rate of partial or complete loss of independence is 17.8–37% in survivors [2–4]. Different from intertrochanteric fracture that has a better bone healing and surgical outcome, femoral neck fracture has a 3–5% rate of avascular necrosis of the femoral head (ANFH) following internal fixation [5] and a considerate proportion of them necessitate hip arthroplasty. Due to coexisting medical conditions (advanced age, blood viscosity), comorbidities (hypertension, heart disease, peripheral vascular disease, cerebrovascular disease) or risk factors (trauma, surgery, limb immobilization), patients with a femoral neck fracture are predisposed to developing venous thromboembolism events, which would further aggravate the already poor prognosis.

Deep venous thrombosis (DVT) and pulmonary embolism (PE) are both important clinical manifestations of venous thromboembolism (VTE). Particularly, the DVT was found to be present in 19.5–32% of patients preoperatively [6, 7] and up to 56% of patients postoperatively [7]. Furthermore, DVT is a major resource of PE [8]. Therefore, routine screening for detection of DVT and targeted therapeutic inventions should be considered to reduce the occurrence of fatal PE. The current guidelines primarily focus on prevention of postoperative DVT in major orthopaedics surgeries (hip or knee arthroplasty, or hip fracture repairs), but pay less attention on the preoperative DVT or DVT at admission. Differing from Europe and the United States where hip fracture surgeries could be implemented within 24-48 h of injury [9], in China it may take several days to get a hip fracture patient to the operation room. Therefore, it is predictable that the incidence of preoperative DVT may be much higher in Chinese patients with hip fracture. In a previous study, Song et al. [10] found two thirds of patients diagnosed with postoperative DVT had already had thrombus before surgery. If patients who have already developed a DVT could be identified immediately at their admission and accordingly early therapeutic rather than prophylactic interventions are given, the surgical outcome or the prognosis might be different.

By far, review of the literature showed scarce data on the preoperative DVT after femoral neck fractures in the elderly, and these studies generally could not provide detailed information on DVT occurrence timing and locations [7, 11]. Accordingly, we conducted this study, with aims to 1, estimate the incidence rate of preoperative DVT; 2, to describe the characteristics of DVT, including the location and the timing; and 3, to investigate risk factors independently associated with DVT.

## Methods

This was a retrospective study and conducted in accordance with the Helsinki Declaration and following the Strengthening the Reporting of Cohort Studies in Surgery (STROCSS). From January 2016 to October 2019, patients 60 years or older who were admitted due to femoral neck fracture and had preoperative examination for DVT of the bilateral lower extremities were deemed to be eligible. The exclusion criteria were: high-energy traumatic fractures, open fractures, pathological or metastatic fractures, multiple fractures, old fractures (>21d from injury), patients with cancer of any site, history of VTE, past peripheral vascular disease, recent anticoagulant therapy (such as aspirin, warfarin, heparin, low molecular weight heparin or others), use of lower extremity compressive devices after injury, no documentation of any DVT examination, and patients with incomplete medical records.

### Diagnosis and classification of DVT

DVT was diagnosed according to the Guidelines for the Diagnosis and Treatment of Deep Vein Thrombosis (3rd edition) proposed by the Chinese Medical Association [12]. Duplex ultrasonography (DUS) scan of bilateral lower extremities veins (common femoral vein, superficial femoral vein, deep femoral vein, popliteal vein, anterior tibial vein, posterior tibial vein and peroneal vein) was performed to determine whether there are DVTs. The diagnostic criteria for DVT were loss of or non-compressibility of the vein, lumen obstruction or filling defect, lack of respiratory variation in above-knee vein segments and inadequate flow augmentation to calf and foot with compression maneuvers. According to our policy, patients with major trauma, especially the elderly hip fracture, should be examined immediately after admission to detect the potential DVTs of the bilateral lower extremities. On basis of the examination results, therapeutic or prophylactic thromboembolic agents are prescribed, and then for about every 3–7 days second or more DUS scans are conducted to detect DVTs on their risk stratification of DVT, until the operation.

Thrombi either in anterior tibial vein, posterior tibial vein, peroneal vein or combined any of them are classified as distal DVT, while in popliteal vein or proximal classified as proximal DVT. Patients with both proximal and distal DVT were considered as the proximal DVT group.

We did not include isolated thrombosis that was solely in the superficial veins (great or lesser saphenous vein) or intramuscular veins (e.g. soleal or gastrocnemius vein), due to their less clinical importance [13].

### Data collection

The inpatient medical record and blood examination reports were retrieved for collecting the relevant data. The detailed information was related to demographics (age, gender and place of residence), body mass index (BMI, calculated by body weight divided by height square), smoking, drinking alcohol, comorbidities (hypertension, diabetes, chronic heart disease, cerebrovascular disease, lung disease, liver disease, renal insufficiency, peripheral vascular disease, etc), injury related data (fracture type based on Garden classification, time from injury to admission and to DUS scan) and the laboratory examination results (platelets, fasting glucose, total protein, serum albumin, red blood cell (RBC), hemoglobin, white blood cells (WBC), neutrophils, lymphocytes, D-dimer, Alanine transaminase (ALT) and aspartate transaminase (AST)).

Obesity was defined as BMI above 28.0 kg/m^2^, accoring the standard suited for the Chinese populations [14]. The comorbidities in the inpatient records were identified by one-by-one check of the primary and secondary discharge diagnoses, always documented at the final page of the inpatient records, to avoid the possible misdiagnosis, inaccuracy or inconsistence. Smoking and alcohol were identified by the patients’- self-reported current smoking or alcohol status after they were admitted, regardless of the frequency or volume, or the lasting years.

### Statistical analysis

We defined patients with preoperative DVT as case group and those without DVT as control group. Continuous variable was expressed by mean and standard deviation (SD), and the difference between both groups was evaluated by the student-*t* test. Categorical variable was expressed as number and percentage, and was tested by the *Chi-square* test or Fisher’s exact test.

Age, BMI, PLT count, plasma D-dimer level and serum albumin level had been demonstrated to be associated with DVT in literature, but the cut-off value for each of them was greatly variable in different settings. Considering that, we reconstructed the receiver operating characteristic (ROC) curve to address the optimal cut-off value for each variable, when Youden index (i.e., sensitivity + specificity-1) was maximized. The area under the curve (AUC) analysis was used to evaluate their statistical power.

Factors which were tested as statistical level at *p* < 0.1 in the univariate analyses were further entered into the multivariate logistic regression to determine their independent effects on DVT. In this process, stepwise backward elimination method was used, and variables retained in the final model satisfied the statistical level *p* < 0.10. The correlation strength was indicated by the odds ratio (OR) and its 95% confidential interval (95%CI). All the analyses were considered statistically significant when *P* < 0.05. Goodness-of-fit of the final model was evaluated by Hosmer-Lemeshow test, with *p* > 0.05 indicating the acceptable result. Nagelkerke R^2^ value was used to quantify the goodness-of-fit, with greater value indicating the better result. All statistical analyses were performed using SPSS24.0 software package (IBM, New York, USA).

## Results

A total of 980 elderly patients with femoral neck fracture were included, including 310 males and 670 females, with an average age of 72.5 ± 8.5 years (range, 60–96 years). According to Garden classification, 181 were classified as type I fractures, 557 as type II and 242 as type III fractures. The average time from injury to admission was 1.4 ± 1.7 days, to DUS scan was 4.2 ± 3.8 days, and to the definite operation was 5.4 ± 3.4 days.

Sixty-seven patients were diagnosed to have DVTs, representing an incidence rate of 6.8% (95%CI, 5.3 to 8.4%), with proximal DVT of 1.7% (95%CI, 0.9 to 2.6%) and of distal DVT of 5.1% (95%CI, 3.7 to 6.5%). The average time from injury to DUS scan for diagnosis of DVT was 6.0 ± 4.7 days. Among DVT patients, 97 thrombi were found, with 1.4 in each patient. Most thrombi were located in distal veins (80.4%, 78/97) and 19 (19.6%) in proximal veins. In 76.1% (51/67) of patients, DVT occurred in the injured extremity, 9.0% (6/67) in the bilateral and 14.9% (10/67) in the uninjured extremity. All of these DVTs were clinically asymptomatic, except 3 case with slight redness of skin but without any complaint.

**Fig. 1:**
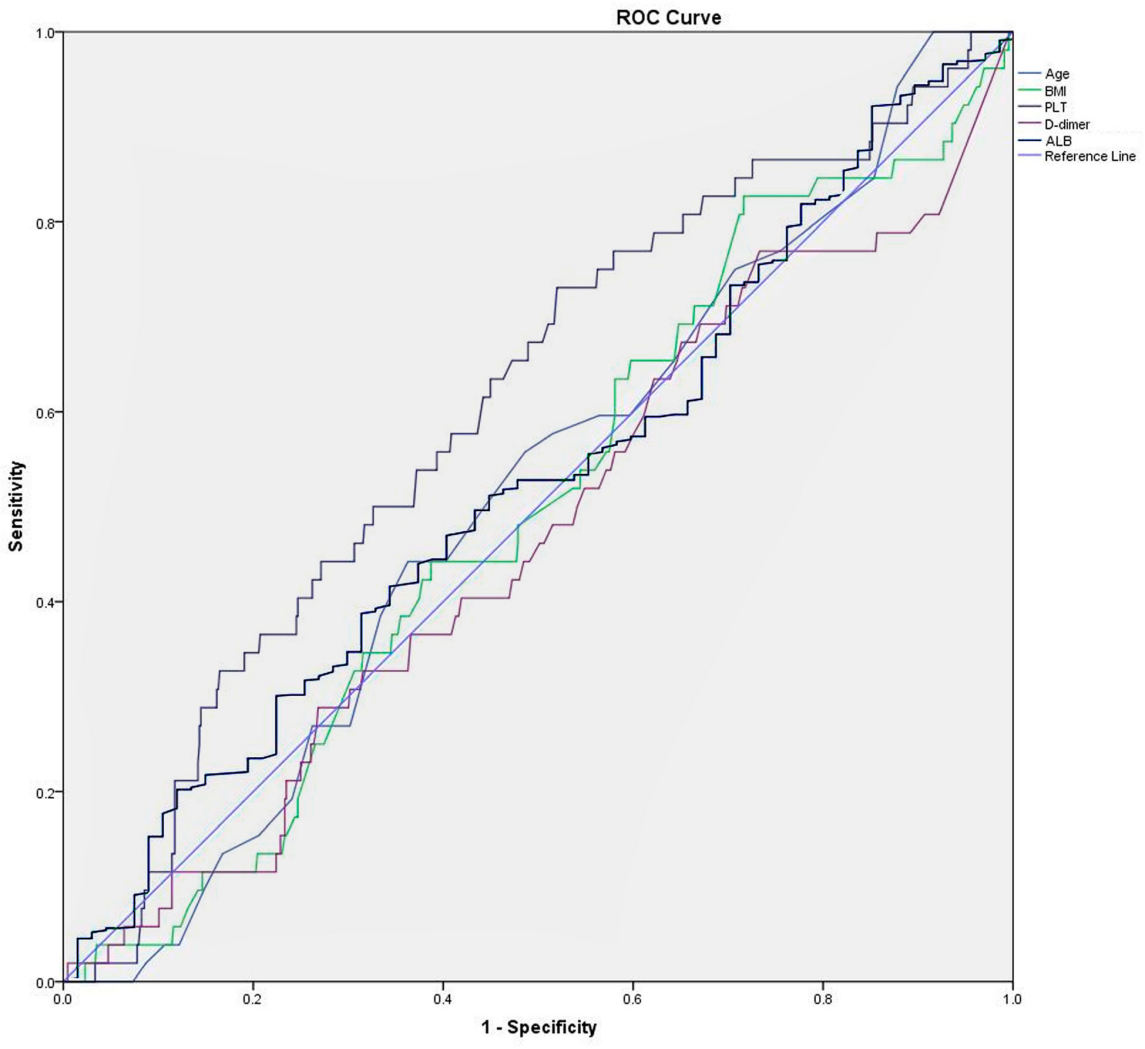
Horizontal axis indicated the 1-specificity and vertical axis indicated the sensitivity of each variable in predicting DVT. The lines in different colors represented the different variables. The area under the curve (AUC) represented the respective ability to discriminate the DVT cases

The optimal cut-off value of PLT was determined to be 220*10^9^/L, tested as statistically significant in the AUC analysis (AUC, 0.605; 95%CI, 0.528 to 0.683; *p* = 0.011). But for other variables, no significant discrimination or inflection point could be determined, with non-significant result in the AUC analysis (*p* > 0.05) (Fig. 1 and Table 1).

**Table 1:** The ROC and AUC to determine the optimal cut-off value for each variable

Variable	AUC	95%CI		*p*
		lower limit	upper limit	
Age	0.512	0.437	0.587	0.779
BMI	0.498	0.420	0.575	0.956
D-dimer	0.469	0.386	0.552	0.456
ALB	0.526	0.456	0.596	0.482
PLT count	0.605	0.528	0.683	0.011

Abbreviation: *ROC* Receiver operating characteristic, *AUC* Area under the curve, *BMI* Body mass index, *ALB* Albumin, *PLT* Platelet. AUC indicated the ability of each variable to evaluate the outcome variable, with a size range of 0 to 1

Patients with DVT and those without DVT significantly differed in term of prevalence of chronic renal insufficiency (16.4% vs 7.3%, *P* = 0.008), current smoking status (28.4% vs 16.2%, *P* = 0.011), time from injury to DUS (6.0 ± 4.7 vs 4.1 ± 3.7, *P* = 0.003) and to definite operation (7.3 ± 3.8 vs 5.0 ± 2.9, *P* < 0.001), the total hospital stay (19.1 ± 7.8 vs 5.0 ± 2.9, *P* < 0.001), prevalence of elevated ALT (19.4% vs 9.4%, *P* = 0.030) and AST (25.4% vs 13.3%, *P* = 0.006), increased count of LYM (37.3% vs 53.5%, *P* = 0.011) and PLT (56.7% vs 36.7%, *P* = 0.001) (Table 2). Regarding other variables, we did not observe the significant results (all *P* > 0.05). In the multivariate analysis, the above variables were entered (chronic renal insufficiency, current smoking status, time from injury to DUS, prevalence of elevated ALT and AST, increased count of LYM and PLT, and in the final model chronic renal insufficiency (OR, 3.37; 95%CI, 1.57 to 7.28), current smoking status (OR, 2.42; 95%CI, 1.23 to 5.63), time from injury to DUS (OR, 1.26; 95%CI, 1.07 to 1.61) and PLT > 220*10^9^/L (OR, 1.94; 95%CI, 1.31 to 3.77) remained significant for association with occurrence of DVT (Table 3). The Hosmer-Lemeshow test showed the adequate goodness-of-fit of the final model (*X*^2^ = 0.375, *p* = 0.639; Nagelkerke R^2^ = 0.472).

**Table 2:** Univariate analysis of factors associated with preoperative deep venous thrombosis (DVT)

Variables	Number (%) of patients without DVT (***n*** = 913)	Number (%) of patients with DVT (***n*** = 67)	***P***
**Gender (males)**	282 (30.9)	22 (32.8)	0.739
**Age**	72.5 ± 8.6	72.5 ± 7.2	0.860
60–69	385 (42.2)	27 (40.3)	
70–79	311 (34.1)	28 (41.8)	
≥ 80	217 (23.8)	12 (17.9)	
**Living place (rural)**	483 (52.9)	39 (58.2)	0.401
**Body mass index (BMI)**	23.6 ± 3.7	23.5 ± 3.4	0.199
18.5–23.9	411 (45.0)	27 (40.3)	0.208
< 18.5	89 (9.7)	8 (11.9)	
24.0–27.9	313 (34.3)	29 (43.3)	
≥ 28.0	100 (11.0)	3 (4.5)	
**Hypertension**	453 (49.6)	36 (53.7)	0.516
**Diabetes mellitus**	209 (22.9)	18 (26.9)	0.457
**Liver disease**	34 (3.7)	1 (1.5)	0.342
**Heart disease**	237 (26.0)	19 (28.4)	0.666
**Rheumatoid arthritis**	42 (4.3)	4 (6.0)	0.515
**Pulmonary disease**	57 (6.2)	2 (3.4)	0.279
**Renal insufficiency**	67 (7.3)	11 (16.4)	0.008
**Cerebrovascular disease**	274 (30.0)	17 (25.4)	0.423
**History of allergy**	14 (20.9)	194 (21.2)	0.946
**Alcohol consumption**	189 (20.7)	17 (25.4)	0.618
**Current smoking**	148 (16.2)	19 (28.4)	0.011
**Previous operation in any site**	272 (29.8)	24 (35.8)	0.300
**Time from injury to DUS (days)**	4.1 ± 3.7	6.0 ± 4.7	0.003
**Total hospital stays (days)**	14.2 ± 6.8	19.1 ± 7.8	< 0.001
**Time from injury to definite operation (days)**	5.0 ± 2.9	7.3 ± 3.8	< 0.001
**Fracture classification**			0.601
B1	166 (18.2)	15 (22.4)	
B2	519 (56.8)	38 (56.7)	
B3	228 (25.0)	14 (20.9)	
**ASA score**			0.368
I-II	605 (66.3)	48 (71.6)	
III-IV	308 (33.7)	19 (28.4)	
WBC (> 10*10^**9**^**/L)**	308 (33.7)	22 (32.8)	0.811
NEU (> 6.3*10^**9**^**/L)**	493 (54.0)	34 (50.7)	0.575
LYM (< 1.1*10^**9**^**/L)**	488 (53.5)	25 (37.3)	0.011
PLT (> 220*10^**9**^**/L)**	335 (36.7)	38 (56.7)	0.001
**RBC (<**Lower limit**)**	578 (63.3)	39 (58.2)	0.149
**HGB (**<Lower limit**)**	523 (57.3)	35 (52.2)	0.392
**HCT (**<Lower limit**)**	581 (63.6)	40 (59.7)	0.437
**PDW (< 12.0%)**	52 (5.7)	2 (3.0)	0.550
**D-dimer (> 0.5 mg/L)**	610 (66.8)	47 (70.1)	0.575
**TP (< 60 g/L)**	454 (49.7)	36 (53.7)	0.655
**ALB (< 35 g/L)**	478 (52.4)	38 (56.7)	0.490
**ALT (> 50 U/L)**	86 (9.4)	13 (19.4)	0.009
**AST (> 40 U/L)**	121 (13.3)	17 (25.4)	0.006
**GGT (> 60 U/L)**	107 (11.7)	9 (13.4)	0.907
**TC (> 5.2 mmol/L)**	48 (5.3)	5 (7.5)	0.571
**TG (> 1.7 mmol/L)**	68 (7.4)	4 (6.0)	0.752
**Sodion (< 135 mmol/L)**	349 (38.2)	26 (38.8)	0.875
**Chloridion (< 98 mmol/L)**	73 (8.0)	7 (10.4)	0.634
**FBG (> 6.1 mmol/L)**	460 (50.4)	39 (58.2)	0.345

**Abbreviations**: *WBC* White blood cell, *NEUT* Neutrophil count, *LYM* Lymphocyte, *RBC* Red blood cell, reference range: Female, 3.5–5.0*10^12^/L; males, 4.0–5.5*10^12^/L, *HGB* Hemoglobin, reference range: Females, 110-150 g/L; males, 120-160 g/L, *HCT* Hematocrit, reference range: Females, 35–45%; males, 40–50%; *PLT* Platelet, *MPV* Mean platelet volume, *PDW* Platelet distribution width, *TP* Total protein, *ALB* Albumin, *ALT* Alanine transaminase, *AST* Aspartate transaminase, *GGT* γ-glutamyltransferase, *TC* Total cholesterol, *TG* Triglycerides, *FBG* Fasting blood glucose

**Table 3:** Risk factors associated with DVT, by univariate and multivariate analysis

Variables	Univariate analyses		Multivariate analyses	
	Crude OR and 95%CI	***P***	Adjusted OR and 95%CI	***P***
Chronic renal insufficiency	2.48 (1.24–5.00)	0.008	3.37 (1.57–7.28)	< 0.001
Current smoking	2.05 (1.17–3.58)	0.011	2.42 (1.29–5.63)	0.004
Delay to DUS (in each day)	1.23 (1.05–1.62)	0.003	1.26 (1.07–1.61)	0.029
Platelet count (> 220*10^9^/L)	2.02 (1.27–3.73)	0.001	1.94 (1.31–3.77)	0.021
Lymphocyte count (< 1.1*10^9^/L)	1.93 (1.16–3.22)	0.011	1.76 (0.89–3.06)	0.097
ALT (> 50 U/L)	2.32 (1.22–4.41)	0.009	1.69 (0.92–4.02)	0.123
AST (> 40 U/L)	2.23 (1.24–3.99)	0.006	1.58 (0.83–3.32)	0.172

Abbreviation: *DVT* Deep venous thrombosis, *DUS* Duplex ultrasonography, *ALT* Alanine transaminase, *AST* Aspartate transaminase, *OR* Odd ration, indicates the association intensity of DVT with each variable

## Discussion

Adequate evidences have shown the risk of DVT is increased immediately after trauma, and is particularly high in elderly patients with hip fracture due to their poor immune status, prevalent underlying disease and stress response to trauma [15, 16]. In this study, we found that the incidence of preoperative DVT following femoral neck fracture was 6.8%, with 1.7% for proximal and 5.1% for distal DVT. Chronic renal insufficiency, current smoking status, time from injury to DUS and PLT > 220*10^9^/L were identified as independent factors associated with DVT.

Previous studies reported the greatly variable incidence rates of DVT following hip fracture, ranging from 2.6 to 35.0% [8, 11, 16–19], mainly dependent on the different settings (pre- or postoperation), participating subjects, definition of DVT, methods used to diagnose the DVT, and particularly whether or not use of thromboembolic agents. Shin et al. [17] reported the incidence of preoperative DVT of 5.8% in patients with a hip fracture, with a comparable time from injury to diagnose DVT (7.6 verse 6.0 days) using CT scan. In a South Korean prospective study, Cho et al. [8] reported the lowest incidence rate of DVT of 2.6% in 152 geriatric patients who were examined by ultrasonography or CT scan, and they attributed this to the earlier admission to hospitalization (90.1% within 3 days after injury). We found the prevalence of DVT following femoral neck fracture in the elderly patients was 6.8%, which was lower than most previous reports but higher than that reported by Shin et al. [17] and Cho et al. [8]. This figure should be cautiously treated under our pre-setting conditions. First, we only focused on femoral neck fracture, with onset age 5–10 years younger than that of intertrochanteric fracture. Second, we only included the DVTs that occurred before prophylactic or therapeutic thromboembolic agents were prescribed. Third, patients who had well-established risk factors, such as history of VTE, past peripheral vascular disease or use of lower extremity compressive devices after injury, were excluded from this study. Fourth, we excluded isolated thrombi in intramuscular veins (e.g. soleal or gastrocnemius vein), which were estimated to take a certain proportion (40–77.2%) [16, 19], because of its relatively clinical significance, at least regarding the relationship with preoperative use of therapeutic anticoagulation [19].

Theoretically, admission to hospitalization and performance of operation as early as possible had more advantages in reducing perioperative complications and improving the prognosis. However, in China and some eastern countries, it seems unpractical to carry out that. In this study, the time from injury to admission was 1.4 days and to the definite operation was 5.4 days, both of which potentially increase the risk of preoperative DVT. Compared to patients who had no DVTs, those with DVTs had a significantly longer preoperative stay (7.3 vs 5.0 days) or delay to DUS (6.0 vs 4.1 days). In the multivariate analysis, we re-confirm this finding that each-day delay to DUS detection was associated with 21% increased risk of DVT. Review of the literature showed the similar conclusion [8, 15, 17]. These findings highlighted the importance of early detection of DVTs in patients with delay to admission or operation, and future study is necessitated to focus on determining the optimal cut-off point for delay to detection, above which the risk of DVT is significantly increased. We suggest that, consistent with our policy, patients admitted 3 days later after injury should be treated as a high-risk group, and DUS scan is priorly performed.

Although all of the DVTs were asymptomatic, the risk of proximal migration of thrombi even to form PE should not be neglected; and such asymptomatic DVTs should be paid more attention, because they cannot provide suggestive significance [20]. We also found a relatively high proportion of DVTs in the uninjured extremity that was 23.9%, which included 9.0% in the bilateral and 14.9% in the non-fractured extremity. This finding was conflicting with a previous report that DVTs were located solely in the injured extremity [17], but was lower than that of another report that 38.9% of DVTs were located in bilateral or only uninjured extremity. Weiss et al. [21] reviewed 6674 trauma patients and found 14% of the DVTs were located in the uninjured extremity. The authors believed it was not adequate in screening the patients for DUS, especially for those with external fixation devices. The detailed mechanism for DVT occurring in uninjured extremity remains unclear, but at least partly related to reduced systemic hemodynamics or blood hypercoagulable state in the elderly patients [22]. For patients with hip fracture, particularly those carrying one or multiple risk factors, more emphasis should be placed for thrombus detection in the uninjured extremity.

In the present study, we identified four independent risk factors for preoperative DVT in elderly hip fracture patients: the aforementioned delay to DUS detection, chronic renal insufficiency, current smoking status and the platelet count > 220*10^9^/L. Conversely, age, gender, BMI or chronic comorbidities except for renal insufficiency were not significant factors, differing from those in previous reports [7, 8, 15–17]. The possible explanation might be that we excluded the well-established risk factors, such as past VTE episode, peripheral vascular disease, recent anticoagulant therapy for other reasons and use of lower extremity compressive devices, which were demonstrated to be major risk factors and highly related to DVT occurrence. Therefore, supposed that any of them is included, the statistical result is predictably altered. Additionally, higher level of AST or ALT was tested to be significant factors for DVT in the univariate analysis, but not in the multivariate analysis after adjustment for multiple variables. AST or ALT was important biomarkers indexes, most often indicating the degree of damage of hepatocyte; but in this study, they are more likely reflecting the severity of skeletal muscle cell around the fracture site. Therefore, it is possible that, relative to systemic disease (renal insufficiency), bone trauma or related hypercoagulability, damage of skeletal muscle after low-energy-impact femoral neck fracture exerts inadequate effect on development of DVT.

Current smoking status may be a modifiable factor, but it could provide little effect in prevention of preoperative DVT, because the response to trauma and especially the blood coagulation remain at the dynamic peak during the preoperative waiting timeframe (3-7 days of injury) [22]. Chronic renal insufficiency itself, its related complications or the treatment agents (hormonal medications, immunosuppressive agents) all might exhibit the adverse effect on DVT occurrence [23]. Furthermore, in patients with renal insufficiency the pharmacokinetics and pharmacodynamics were affected [24]. Future study is necessary to elucidate exactly which mechanism acts or their effect magnitude in DVT formation. The increased platelet count might be the result of innate immune responses, together with activated coagulation and complement system, which resulted in a stemming hemorrhage and protected against bacteria invasion [22], and imbalanced innate immune response likely causes complementopathy or coagulopathy, resulting in DVT formation. Although most not modifiable, these identified risk factors should be kept in mind when assessing the risk of DVT and thereby the stratifying the patients.

Despite a large sample and multivariate potential factors included for adjustment, this study had several limitations. First, the inherent limitation of the this study was the retrospective design, which may affect the accuracy and precision in data collection and introduce the unavoidable selection bias. Second, as with every other multivariate analysis, we were unable to include all confounding factors, therefore the residual confounding remained. Some potential factors, such as the duration of immobolization of injured extremity and the use of glucocorticoids, were not captured. Third, the relationship between variables and DVT identified in this study was correlative rather than causative, which was decided by the cross-sectional nature of this study. Therefore, these results should be interpreted with caution. Fourth, this is a two-center study, and both participating hospitals are teritiary referral hospitals, which might limit the ablity to generalize these findings to elsewhere.

In conclusion, this study demonstrated that the incidence of preoperative DVT in elderly femoral neck fracture patients was 6.8%. In patients with DVT, 23.9% had thrombi in the uninjured extremity, highlighting the necessity of adequate screening for DVT. Chronic renal insufficiency, current smoking status, delay to DUS and PLT > 220*10^9^/L were identified as independent factors associated with DVT. These data are helpful to understand the epidemiologic characteristics of DVT following hip fracture, and should be kept in mind when in individualized risk of DVT and accordingly the risk stratification.

## Data Availability

All the data will be available upon motivated request to the corresponding author of the present paper.

## Abbreviations

DVT: Deep Venous Thrombosis
DUS: Duplex ultrasonography
ANFH: Avascular Necrosis of the Femoral Head
STROCSS: Strengthening the Reporting of Cohort Studies in Surgery
VTE: Venous thromboembolism
PE: Pulmonary embolism
BMI: Body mass index
ROC: Receiver operating characteristic
AUC: Area under the curve
RBC: Red blood cell
WBC: White blood cell
ALT: Alanine transaminase;
AST: Aspartate transaminase
OR: Odd ratio
CI: Confidential interval
SD: Standard deviation
